# Concept of older person autonomy: phenomenological study of the opinion of specialist nurses

**DOI:** 10.1097/j.pbj.0000000000000178

**Published:** 2022-12-01

**Authors:** Andreia Maria Novo Lima, Maria Manuela Ferreira da Silva Martins, Maria Salomé Martins Ferreira, Carla Sílvia Fernandes, Soraia Dornelles Schoeller, Adriana Raquel Neves Coelho, Vítor Sérgio Oliveira Parola

**Affiliations:** a Abel Salazar Institute of Biomedical Sciences, Escola Superior de Saude -Fernando Pessoa, CINTESIS, Porto, Portugal,; b Nursing School of Porto, CINTESIS, Porto, Portugal,; c Instituto Politécnico de Viana do Castelo - Escola Superior de Saúde, UICISA, Portugal,; d Nursing School of Porto, CINTESIS, Porto, Portugal,; e Fe eral, University of Santa Catarina, Brazil,; f Nursing School of Coimbra, UICISA, Coimbra, Portugal.

**Keywords:** personal autonomy, aged, nurses, concept formation, qualitative research

## Abstract

**Background:** The concept of autonomy is composed of several components, making it a multidimensional concept. This includes cognitive ability, intellectual ability, emotional intelligence, social situation, and physical ability skills. This study aimed to describe the experiences attributed to the meaning of the concept of autonomy, by specialist nurses.

**Methods:** A descriptive phenomenological study was carried out, according to the Giorgi method. Eighteen nurses were recruited at a hospital in Portugal. Data were collected through individual interviews.

**Results:** The 18 specialist nurses work on average as nurses for 16years and are specialist nurses; for an average of 5.9years, most are specialist nurses in rehabilitation nursing. From the data analysis, six themes emerged: ability to do, ability to resolve, decision ability, cognitive ability, social integration, and emotional intelligence.

**Conclusions:** Most professionals perceive the concept as the ability to perform activities of daily living and the ability to make decisions, especially concerning informed consent. It is necessary to raise awareness of the breadth of this concept since the quality of life and dignity of the elderly person depends on autonomy.

## Introduction

In the relationship between health professionals and the client, the basic principle of respect for their autonomy is essential. The promotion/maintenance of autonomy using the right of self-determination dignifies and sustains the integrity of the person, conditions that are fundamental for the promotion of quality of life.^1^

It is understood that autonomy implies the duty to respect the decision-making capacity and involves two aspects that complement each other in full, the recognition of the person’s ability to make their own decisions and the promotion of conditions, namely in health interventions, which favor the exercise of that same autonomy.^2^

In the literature, it can be seen that there are differences in the definition of the concept between the various disciplines of knowledge, however, analysing the concept it is possible to perceive that they complement each other, being essential for understanding the scope of the same, especially for the disciplines, with intervention in practice as are the cases of medicine and in particular nursing.^3^

Some authors refer to autonomy as being only the decision-making capacity and compliance with obtaining informed consent^4–6^; she is driven by the motivation to dominate her own being, namely her destiny, to control her own life and behavior.^7,8^ However, it comprises several dimensions: biological, social, psychological, and spiritual, and the integration of all these dimensions is essential when we intend to understand the true essence of the concept.^9,10^

Other authors emphasize, in addition to the aforementioned capacities, the ability to execute^11^ and to act consciously, expressing self-government and the ability to control their own lives, participating in a dynamic between social class, life history, race, gender, and cultural contexts,^12^ where it is inserted, where in most cases power relations persist.^13^

The performance of an autonomous life also depends on the person's perception of himself, including his dignity and his emotions, being necessary effective emotional management.

Autonomy is not limited to executive competence in individual decisions. Its respect cannot be interpreted merely as a simple matter of non-intervention in the decisions taken, as these depend on the person's empowerment and intellectual capacity.^3^ To make autonomous decisions, to deal with health issues and life in general, the person has to have the intellectual capacity; only then can he experience volition and empowerment.^14^

In short, the concept of autonomy presupposes capabilities, namely cognitive, intellectual, physical and social capacity, and emotional intelligence.^3,7,9,13,15^

In the context of the provision of nursing care, the term of autonomy is seen daily in the speeches of most professionals, wanting to refer to the physical independence of the user, so it is important to understand effectively what the perception that specialist nurses hold regarding the concept of autonomy.^16^

This aspect assumes particular importance when approaching this concept in the area of the older person, as these, due to the processes resulting from ageing and culture, make them vulnerable people, thus placing them at risk of experiencing immobility processes. These immobility processes call into question not only independence, but also autonomy, since they affect the respiratory, cardiovascular, gastrointestinal, urinary, metabolic, nervous systems, skin and integuments and musculo-skeletal systems, referring the person to the social isolation and cognitive commitment.^17^

This study aims to: describe the experiences attributed to the meaning of the concept of autonomy by specialist nurses. To answer the following research question: What is the meaning of the clinical experience of specialist nurses concerning the concept of autonomy for the older person?

## Methods

A qualitative study of a phenomenological approach was chosen to understand the experiences attributed to the meaning of autonomy by specialist nurses. Phenomenology describes a phenomenon through the sense and meaning people attribute to their experiences, using its analysis.^18^

The methodological orientation used was the phenomenologi-cal analysis of Giorgi,^19^ whose objective is to search for the meanings of human action through analysis and understanding.

Data collection was carried out in two health institutions located in the north of Portugal: a medium-sized general hospital with about 180 beds and an intensive care unit; and a large and highly complex General Hospital, which acts as a reference for the region.

For its realisation, authorization was requested from the Institutions' Board of Directors. Within the scope of the application of data collection instruments, all ethical procedures were respected, by signing the Informed, Free and Informed Consent Term, as well as obtaining approval by the Ethics Committees of the two Institutions (Opinions No. 324/17 and No. 11/18), before the respective harvest. This study also conforms to the Consolidated Criteria for Reporting Qualitative Research guidelines.

Data were collected between March and December 2018. The study inclusion criteria considered the fact that specialist nurses have worked with the older person for at least 6 months, this period ensures that the integration of the professional in the institution is already accomplished, and are holders of the specialties of Rehabilitation Nursing, Medical-Surgical Nursing, Mental Health Nursing and Psychiatry and Community Nursing, and to develop their professional activity in hospitals and/or in the community. All generalist nurses, specialist nurses with the specialities of Child Health Nursing and Pediatrics and Maternal and Obstetric Health Nursing were excluded.

Nurses who met the recruitment requirements were identified, with the help of nurse managers, using intentional non-probability sampling. After this contact, one of the authors sought out the respective specialist nurses, obtaining their consent and informing orally and in writing the objectives and procedures of the study. A total of 18 specialist nurses working in internal medicine, surgery, and orthopaedics units met the inclusion criteria and were therefore invited to participate in the study. All invited nurses agreed to participate in the study, signing the Free and Informed Consent.

The interviews were conducted individually, with a duration of approximately 30 minutes and were carried out in a private room in the unit where they provided care. The data were collected through a semi-structured interview, as this technique facilitates the narrative.

The data collection instrument was made up of two parts. The first included a sociodemographic questionnaire to characterise the participants, and the second, a semi-structured script, with open questions that pointed to the report of their opinion and lived experiences. The questions were asked to promote the participants’ reflection and started with the following question: “What is autonomy for you?” and “How does it characterise the autonomy of an older person?”.

Data were collected by one of the authors. The data were saturated after 14 interviews. However, 4 more interviews were made, since they are already scheduled with the participants. All interviews were audio-recorded and transcribed in full. The interviewer made the transcripts. The interpretation of the information obtained was carried out by two of the researchers, with high degree of consensus, in the present study, based on the Giorgi method, according to the four steps, which the author recommends: (1) obtaining the meaning of the whole, using the full reading of all responses, in order to obtain a general idea of the lived experience; this was done without a critical reflection on the experience—the researcher has an attitude of phenomeno-logical reduction; (2) discrimination of units of meaning: the transcribed interviews were re-read for a long time, and when a transition of meaning was identified in the transcripts, they were marked as relevant aspects of the phenomenon under investigation (still expressed in the common language of the participants); (3) transformation of the common language of the units of meaning into scientific language: the units of meaning were rewritten in language appropriate to the phenomenon under study, remaining faithful to the meanings expressed by the participants. This imaginative variation allowed us to determine the essence of the phenomenon’s structure; and lastly (4) synthesis of the transformed units of meaning, in a descriptive structure of the experience lived by specialist nurses.^19^

The present study adhered to credibility, dependability, transferability, and confirmability for sustaining rigor.^20^

To achieve credibility, during the interviews, cross-checks were performed to clarify and confirm the information mentioned by the participants. The interviewer was a Doctor of Philosophy Student with more than 10 years’ experience as a nurse who is trained and experienced in qualitative research. To avoid influencing the results, she tried to avoid referring to her own experiences and notions and instead let the participants express their own thoughts.

Dependability was achieved by describing the characteristics of the participants, the selection process, and description of the steps and results of the research. The second author also conducted an individual analysis to provide rigor to the process. The degree of consensus was higher; any disagreements that arose between the authors were resolved through discussion.

A detailed description of the characteristics of the participants in the study and the context of the study was provided, providing that readers can determine if the results obtained can be transferred (transferability) to another context.

**Table 1:** Demographic characteristics of the participants (n = 18)

Characteristics
Gender, n	
Male	17
Female	1
Age, y	Range, 32–60
	Mean, 39.6
Years of professional experience, y	Range, 7–40
	Mean, 16.3
Years of professional experience as a specialist, y	Range, 0.5–12.0
	Mean, 5.9
Specialists, n	18
Speciality area, n	
Rehabilitation nursing	14
Community nursing	2
Medical-surgical nursing	2

Finally, to achieve confirmability, the interviews were conducted by the first author, who is not a staff member at this hospital and shared no relationship with the participants.

Atlas-ti software version 8.4 was used to systematize and catalog the analyzed material. The data obtained were coded with the letter “E” and a number was assigned to each participant.

## Results

Of the 18 specialist nurses who participated in the present study, as can be seen in Table 1, the majority are female (n = 17), with an average age of 39.6years, working on average as nurses for 16 years, for an average of 5.9 years, most are specialist nurses in rehabilitation nursing (n = 14; 77.8%).

The results that emerged from the recordings’ analysis were organised by themes.

**Figure 1:**
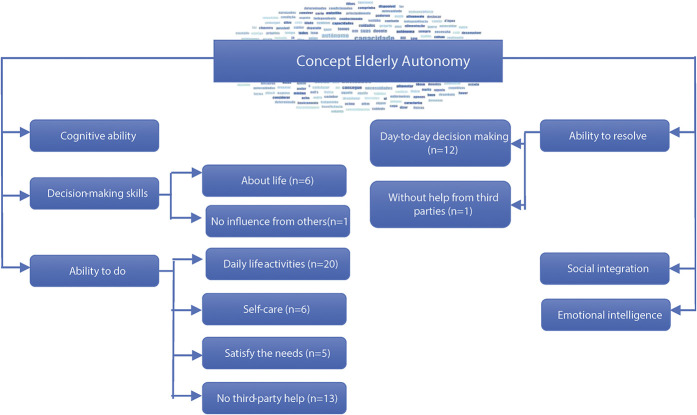
Conceptual representation of the themes and subthemes based on the findings.

The analysis of the data resulted in six themes that reflect the opinion and experiences of specialist nurses, concerning the concept of autonomy for the older person: ability to do, ability to resolve, decision ability, cognitive ability, social integration, and emotional intelligence as organised in Figure 1. These themes are then explored using meaningful text captured from the participants’ narratives.

When questioned by specialist nurses about what autonomy is and how it characterizes the autonomy of an older person, most associate this concept with the ability to do, namely activities of daily living, without the help of third parties, with the ability to resolve, especially in which concerns decision-making on a day-to-day basis, with less reference to cognitive ability, decision-making capacity, social integration, and emotional intelligence.

It is recognized that the concept of autonomy is driven by an intrinsic source of motivation to dominate being, being and its destiny, referring to the freedom to live and the quality of life.

### Ability to do

Related to executing autonomy, the ability to do, is attributed to the competence and freedom to carry out the tasks that you intend to perform.

In the participants’ discourse, the association of the concept of autonomy with the ability to carry out activities of daily living is evident, when they refer: “ … *autonomy* … *is having the ability to carry out activities of daily living independently”* [E6], “is *to satisfy the needs of daily life … in activities of daily living”* [E7], “*is the ability that the individual has to perform activities of daily living”* [E9], “*is a person who after hospital-isation you can do your hygiene, you can … walk, you can cook … ”* [E12], they are “*the habits you have in your daily life”* [E14], “*autonomy … is based on activities of daily life, mainly in food, hygiene care, using the toilet, walking”* [E14], *“Autonomy is the ability of the individual or person … to do their daily activities”* [E15], and “*Autonomy for me is the ability that people have … to make their lives, to be independent … on a physical level”* [E18].

### Ability to resolve

Another aspect that stood out in the speech of specialist nurses was the ability to resolve, relating this to the ability to make decisions on a daily basis and without the help of third parties: *“Person’s autonomy is when they are able to solve their daily situations without the help of others”* [E1] and “*autonomy for me is the ability that people have to make their lives … manage your own life”* [E18].

### Decision-making skills

As mentioned by the participants, decision-making autonomy refers to the ability and freedom to make decisions. Thus, from the theme of decision-making skills, the following subthemes stood out: without influence from others and on life, which can be seen through the speeches: “*Many times people are conditioned in their own autonomy, that is, they are influenced by some physical, psychological aspect, of the environmental context, of the social context, which influence their ability … ”* [E2], to the full satisfaction of autonomy *" the user must be able to decide and decide what is best for him … must be involved in the decision … must have knowledge and through knowledge, have autonomy to … decide what he really wants … ”* [E4], and “*for me autonomy is the person being able to decide for themselves, the life path … getting themselves, making decisions about social, physical, psychological life … everything, be it to decide everything”* [E5].

### Cognitive ability

To control one’s own life and behavior, as highlighted, it is crucial that the person has cognitive skills, which enable him for literacy and empowerment, thereby enabling it to freedom of choice and control of its actions. The participants described the theme of cognitive ability as the “*ability to assimilate what the professional intends to do”* [E2], autonomy “*clearly depends on the person's cognitive ability”* [E8],and *"mental capacities … ”* [E14], is having “*their cognitive abilities to know how to say, I want this or I want that”* [E17], and “*it is the ability that people have … to make their lives, to be independent … want physically or mentally”* [E18].

### Social integration

Participants mention that social, economic, and professional situations are decisive factors for decision-making capacity.

From the theme of social integration, as can be seen from the grafts: the person’s autonomy is influenced by “ *… the very environmental context, the very social context, which influences their capacity”* [E2], a person autonomous manages “*herself, to make decisions about … social, physical, psychological life"”* [E5], is the ability “*to go to the cafe, to socialise”* [E15], and “*to relate to her friends, this is what characterises his autonomy, is to be able to be active at all levels”* [E18].

### Emotional intelligence

In the performance of an autonomous life, the person’s perception of himself is crucial, including in this perception his emotions and self-knowledge, which presuppose the need for optimal emotional intelligence. This ability is evidenced through the speeches of specialist nurses: autonomy “is influenced by some physical, psychological aspect” [E2].

## Discussion

The sociodemographic and professional data of the sample corroborate the data from the Portuguese Nurses Association,^21^ in which the majority of nurses are female, the age groups that comprise the largest number of nurses are between 31 and 35 years old, and 36 and 40years old, with 13,607 and 13,164 nurses, respectively, the majority being specialist nurses in Rehabilitation Nursing. The efficiency and effectiveness of decision-making in nursing depends on professional experience, which is considered an essential aspect for the realization and deepening of knowledge and evidence-based practice.

The various disciplines have contributed to the evolution of the concept of autonomy over time, namely medicine, nursing, ethics, bioethics, psychology, philosophy, and sociology. Thus, the concept has been acquiring broader limits, which should be known and put into practice, by those who are largely responsible for the processes of acknowledging/accrediting qualifications, nurses, and especially specialist nurses, as holders of specialized knowledge and supported. Here nursing, as a profession and discipline, assumes particular importance.^22^

In the speeches, it is notorious, professionals associate the concept of autonomy of the older person with the ability to execute and the need for people to make their decisions on a daily basis and to act consciously, expressing their cognitive ability, through self-government and control of their own life,^4,23,24^ these being the most highlighted aspects throughout the speeches of specialist nurses. These data corroborate the study carried out by Bennett et al,^15^ in which empowerment is seen as an integral component for the practice of care, particularly when the intention is to improve the quality of life and therefore, the person's autonomy and for this purpose, it is necessary that the person has cognitive capacity. More practical areas, such as nursing and medicine, define the concept as physical and cognitive capacity for conscious decision-making, while areas such as sociology, philosophy, ethics, bioethics, and psychology, add intellectual capacity, emotional intelligence, and social situation.^3^ Therefore, it is urgent that nurses understand the concept of autonomy as a whole, because only then they will be able to give adequate answers to the needs of the people they care for, particularly the older person, since these by the specific characteristics, proper to the process of aging, are subject to immobility processes, which jeopardise not only independence, but autonomy.^25^

In the speeches of specialist nurses, an autonomous older person is the one who has the ability to decide on his own life and with that ability to solve his problems, corroborating the results of Lamine et al,^14^ who stress in their study that autonomy presupposes the intellectual capacity for conscious decision-making, as a result of the need to feel volitional and in possession of their own actions and Kirkscey,^26^ when he mentions that the person can only be considered autonomous if he experiences the empowerment and competence to deal with issues related to his life.

The participants allude in their speeches to the importance of emotional intelligence and social integration, in a subtle way, and these results do not corroborate the study of Krishna et al,^12^ and Henry et al,^27^ as these refer in their study that the person’s autonomy depends directly on the social environment, marked by the economy, politics, ethnicity, gender, culture, self-knowledge, and the ability to manage emotions according to the circumstances of life, being subject to the integration of all these factors.

Knowledge of concepts and their limits is essential for the person's dignity and quality of life, assuming particular importance in clinical practice,^22^ since they are areas where knowledge in the health field is applied and where they effectively assume importance.

Nursing practices with the person depend on the concepts involved in the care process,^10,22^ therefore, there is a need for a conceptual change/deepening to integrate a systematized view of the concept of autonomy in the elderly, where it assumes particular importance, due to the vulnerability these people face as a result of changes in biological, physiological, and social processes.^17^

The results of this study demonstrate a great approximation between theory and clinical practice, with regard to nursing, as all consulted studies in the field of nursing, which address the concept, emphasize physical capacity and decision making as factors important in promoting autonomy.

Qualitative research, originating from the human and social sciences, allows deepening the knowledge about the phenomena. This methodology allows a greater approximation of the phenomena, facilitating its knowledge integrally and profound-ly.^19,28^ For the present study, the qualitative methodology made it possible to know in the nurses’ perception the concept of autonomy, facilitating the understanding of the phenomenon that comes from here, that is, the way these nurses work in practice the autonomy of the older person. Like Watson^22^ stresses, the concepts are the foundations of nursing practices, so they must be well defined and based on a sustained theoretical construct.

## Conclusion

The results of the present study guide and stimulate the need for a closer look at the concept of autonomy on the part of nurses since they are the great mentors of its promotion and maintenance in clinical practice. In this sense, it was possible to understand how nurses perceive autonomy concept in the older person through the investigation. Using the phenomenological method, this by the potential of participation and reflection, allowed to expand the knowledge about the concept of autonomy of the older person, perceived by specialist nurses.

Regarding clinical implications, given the ageing population, more and more nurses will contact older persons in various contexts in the future. Therefore, acknowledging these experiences is crucial because it forces nurses, other health care professionals, and managers in health areas to reflect on the challenges experienced by these nurses and adopt a proactive attitude to promote elderly autonomy. It is essential to emphasize these aspects in the training of nurses, reinforcing all elements that make up the concept due to the importance of its implementation in practice and research.

The limitations of this investigation focus on the absence of specialist nurses in mental health, which could be interesting for exploring the concept, and the lack of studies, both primary and secondary, carried out on the concept of autonomy. Future investigations must be carried out, including more nursing professionals, in different care contexts for the older person, namely psychiatric contexts.
